# Popularity-Aware Closeness Based Caching in NDN Edge Networks

**DOI:** 10.3390/s22093460

**Published:** 2022-05-02

**Authors:** Marica Amadeo, Claudia Campolo, Giuseppe Ruggeri, Antonella Molinaro

**Affiliations:** 1 DIIES Department, University Mediterranea of Reggio Calabria, 89100 Reggio Calabria, Italy; marica.amadeo@unirc.it (M.A.); giuseppe.ruggeri@unirc.it (G.R.); antonella.molinaro@unirc.it (A.M.); 2 National Inter-University Consortium for Telecommunications (CNIT), 43124 Parma, Italy; 3 Laboratoire des Signaux et Systémes (L2S), CentraleSupélec, Université Paris-Saclay, 91190 Gif-sur-Yvette, France

**Keywords:** Named Data Networking, information centric networking, caching, edge networks, 6G

## Abstract

By enabling name-based routing and ubiquitous in-network caching, Named Data Networking (NDN) is a promising network architecture for sixth generation (6G) edge network infrastructures. However, the performance of content retrieval largely depends on the selected caching strategy, which is implemented in a distributed fashion by each NDN node. Previous research showed the effectiveness of caching decisions based on content popularity and network topology information. This paper presents a new distributed caching strategy for NDN edge networks based on a metric called popularity-aware closeness (PaC), which measures the proximity of the potential cacher to the majority of requesters of a certain content. After identifying the most popular contents, the strategy caches them in the available edge nodes that guarantee the higher PaC. Achieved simulation results show that the proposed strategy outperforms other benchmark schemes, in terms of reduced content retrieval delay and exchanged data traffic.

## 1. Introduction

A multitude of innovative applications, ranging from holographic telepresence to extended reality (XR), are expected to be delivered on top of sixth-generation (6G) networks, which would highly challenge the existing Internet infrastructure. Disruptive solutions are needed to cope with the demands of such future bandwidth-hungry and low-latency applications. By supporting name-based routing and ubiquitous in-network caching, the Named Data Networking (NDN) [1] paradigm is identified as a key enabler of 6G network architectures aimed at improving content distribution.

NDN is an information centric networking (ICN) architecture that promotes a communication model directly based on topology-independent content names, instead of internet protocol (IP) addresses. Content retrieval is based on the exchange of two named packets: the Interest, transmitted by the end-clients to retrieve the content, and the Data, transmitted by any node owning a copy of the content. Each Data packet is uniquely named and secured and, therefore, it can be cached by any NDN node in the path between the requester and the original source.

More specifically, NDN nodes are provided with a Content Store (CS) to cache incoming Data packets. The default caching strategy in NDN is Cache Everything Everywhere

(CEE) coupled with Least Recently Used (LRU) replacement, i.e., each node caches each incoming Data packet and, if the CS is full, the LRU policy is applied to remove an existing item and make room for the new one. Although this strategy can speed up the data retrieval, several studies have showed that better performance can be obtained if selective decision strategies are implemented that improve the cache diversity [2].

One of the most important caching decision metrics is the content popularity. Typically, Internet contents show a skewed popularity distribution [3]: only a few contents are highly requested and deserve to be cached. Another crucial caching decision metric is the centrality of the nodes. The work in [4] focuses on the betweenness centrality metric and demonstrates that caching at the most central nodes can increase the cache hit probability and decrease the cache eviction rate, as these nodes are traversed by the majority of content requests. However, from the perspective of content retrieval, the node centrality should be re-defined by taking into account the capability of the node of satisfying the content requests, in addition to its topological feature [5,6].

So far, existing work [5,6,7] defined a popularity-aware betweenness centrality metric to enable coordinated caching decisions between distributed cachers. Basically, these strategies weight the betweenness centrality of a node with the popularity of the contents that can be transmitted through it. By introducing a relevant signalling among the nodes, collaborative decisions are deployed, where the most popular contents are cached in the most central nodes. However, in edge topologies, typically characterized by hierarchical topologies [6,8], the nodes with the highest betweenness centrality are also far away from the end-clients, i.e., the content consumers. Therefore, they cannot guarantee a low retrieval delay and a small network traffic. Instead, it would be crucial to cache the content as close as possible to the consumers to meet the proximity requirements of upcoming 6G applications.

In this paper, we define a new caching scheme based on a popularity-aware closeness (PaC) metric, which allows to cache the most popular contents in the edge nodes according to their proximity to the majority of requesters. Thanks to lightweight signalling piggybacked in Interest and Data packets, the potential cachers are ranked according to the PaC metric, and the best available cacher per path is selected. Performance evaluation shows that the conceived strategy is able to limit the retrieval delay while maintaining low exchanged traffic and the signalling overhead, compared to related literature based on the betweenness centrality metric [6].

The remainder of this paper is organized as follows. Section 2 provides background material on caching strategies in NDN. The proposal is discussed in Section 3 and evaluated in Section 4. Final remarks are reported in Section 5.

## 2. Background and Motivations

### 2.1. NDN in a Nutshell

**Figure 1:**
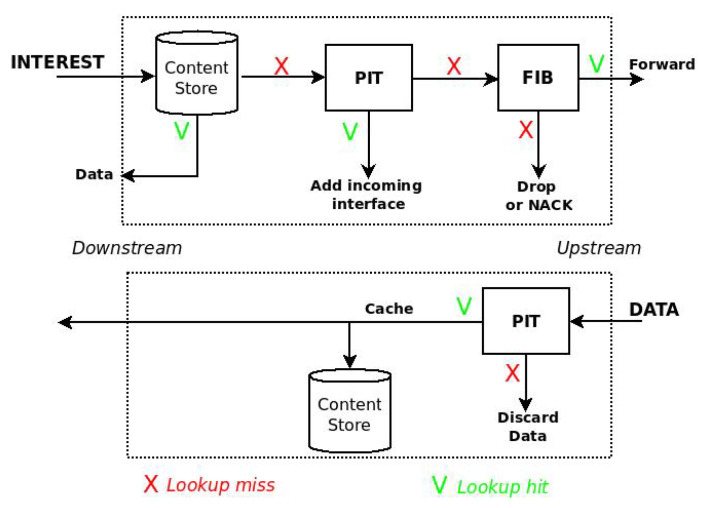
Forwarding process in NDN.

In NDN, caching operations are embedded in the forwarding process of the Interest and Data packets, which is shown is Figure 1.

At the reception of an Interest packet, the node looks in the CS and, in case of a name matching, it immediately sends the Data back. Vice versa, the node checks if an equal request is pending in the Pending Interest Table (PIT) and, in case of a positive outcome, the incoming interface of the Interest is added to the PIT entry and the packet is discarded, thus reducing the traffic in the network. If the PIT matching fails, the Interest is forwarded according to named-based forwarding rules towards the original source.

When the Data packet arrives, the node checks for a name matching in the PIT and, in case of a positive outcome, the packet is cached and then forwarded towards the consumer(s). Vice versa, if the PIT matching fails, the Data is considered unsolicited and it is discarded.

The vanilla NDN caching strategy, CEE, typically leads to poor performance due to the lack of cache diversity [2]. A conventional scheme that limits the cache redundancy without introducing much complexity is Fixed Probability-based caching, where the Data packets are cached according to a fixed probability, usually set to 0.5 [9].

### 2.2. Caching Strategies in the Literature

To improve the content retrieval performance, several NDN caching strategies have been proposed in the last few years [2]. Among them, we can identify two major categories, namely popularity-based schemes and centrality-based schemes, which leverage, respectively, content popularity information and network topology information to select which contents to cache and where.

In popularity-based schemes [10,11,12,13,14], NDN nodes cache the most popular contents according to the locally measured rate of received Interests, which is stored in a Popularity Table. Typically, a threshold based mechanism is considered: contents that are requested a number of times higher than the threshold are cached.

In centrality-based schemes [4], instead, the caching decision depends on the *importance* of the nodes, expressed in terms of topological centrality. For instance, the pioneering proposal called *Betw* [4] leverages the betweenness centrality of the nodes as decision metric and replaces contents with the least recently used (LRU) policy. According to the graph theory, given a set *V* of network nodes, the betweenness centrality ($C_{B}$) of a node $v_{i}\in V$ is defined as:

(1) $$C_{B}\left(v_{i}\right)=\sum_{v_{k}\neq v_{j}\neq v_{i}\in V}\frac{\sigma_{v_{k},v_{j}}\left(v_{i}\right)}{\sigma_{v_{k},v_{j}}},$$

with $\sigma_{v_{k},v_{j}}\left(v_{i}\right)$ and $\sigma_{v_{k},v_{j}}$ being, respectively, the number of shortest content delivery paths from the two endpoints $v_{k}$ and $v_{j}$ that pass through $v_{i}$ and the total number of shortest content delivery paths between the same endpoints. *Betw* strategy is built on the following intuition: if a node $v_{i}$ is traversed by many content delivery paths, then it is more likely to get a cache hit. Therefore, contents are cached in the nodes with the higher betweenness centrality.

In [5], however, the authors observe that the topological centrality metric alone does not reflect the importance of a node from the content delivery perspective. Therefore, the authors define a new popularity-aware centrality metric, which aims at placing the most popular contents at high central nodes, and the remaining contents with decreasing popularity at nodes with decreasing centrality score. With the same target, the Betweenness and Edge Popularity caching (BEP) strategy [6] leverages a coordinated signalling mechanism piggybacked into Interest and Data packets. In BEP, the edge nodes (i.e., the leaf nodes directly connected to the consumers) track the number of received requests and periodically compute the content popularity with an exponential weighted moving average (EWMA) formula. The information is maintained in a Popularity Table, where contents are also ranked in terms of popularity. When a content request arrives, the edge node includes the correspondent popularity ranking in the Interest and forwards it towards the origin source. In addition, the Interest carries an array of betweenness centrality values, which is filled by all on-path routers. When the origin source receives the Interest, it compares the popularity ranking against the available betweeneess values and identifies the cacher by matching the two metrics, i.e., if the content has the highest popularity, it will be cached in the node with the highest centrality, and so on.

Despite their differences, all the noted approaches consider the betweenness centrality in the caching decision. However, this metric does not guarantee the minimum retrieval delay for the consumers. Indeed, especially when considering edge domains, typically characterized by hierarchical topologies [8], the nodes with the highest betweenness centrality are usually far away from the leaf nodes, where the consumers are attached. Instead, a peripheral edge node, e.g., a base station (BS), has typically low betweenness centrality, but it can be very close to the consumers and cover a key role as cacher.

Given a content *x*, the closest node to the requesters of *x* would be able to deliver the content with the lowest delay. Therefore, to take advantage of the limited cache capacity at the edge, it is necessary to select the most popular contents and cache them in the closest nodes along the delivery paths. In addition to reducing the content retrieval latency, caching contents close to the consumers allows for the reduction of intra-domain traffic. Indeed, contents may traverse a lower number of hops and free bandwidth resources over edge links.

Table 1 compares the main features of the related caching strategies available in the literature and our proposal.

## 3. PaC-Based Caching

### 3.1. Main Pillars and Assumptions

To capture the aforementioned needs and overstep the limitations of existing solutions, we propose a different topological metric that accounts for *the proximity of potential cachers to the consumers and is weighted by the content popularity*. We leverage the resulting metric, namely *PaC*, in a new strategy aimed at caching the most popular contents as close as possible to the majority of consumers in order to limit the data retrieval delay and the exchanged data traffic.

**Figure 2:**
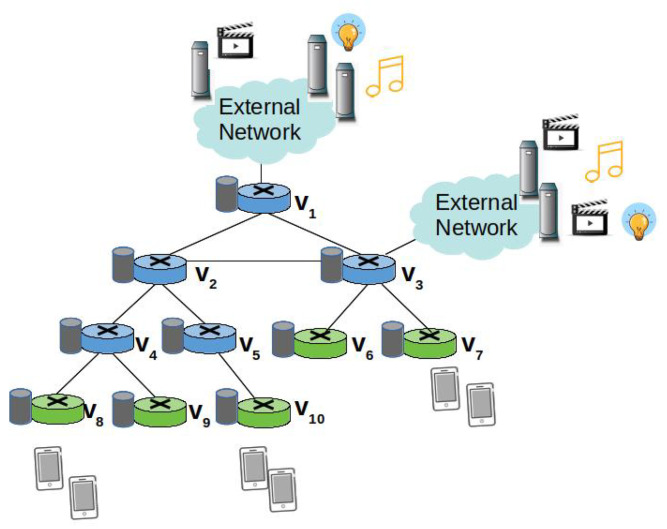
Reference scenario.

As shown in Figure 2, the reference scenario of our study is an edge domain, e.g., the backhaul network of a mobile network operator, a campus network, composed of a set *V* of NDN nodes [8]. A subset of nodes denoted as I⊂V act as ingress nodes that consumers are connected to. A few other nodes, instead, act as egress nodes towards the content sources, i.e., remote servers hosting the contents. A catalogue *X* of cacheable contents is considered.

For ease of reference, the key notations used in the paper are summarized in Table 2.

All the nodes have caching capabilities and implement the traditional NDN forwarding fabric with the *best route* strategy, i.e., Interests are forwarded along the shortest path between ingress and egress nodes. A routing protocol, e.g., named-data link state routing protocol (NLSR) [15], is enabled for intra-domain dissemination of both connectivity and name prefix information.

To enable the PaC-based caching (PaCC), the following main modifications are foreseen to the legacy NDN routines, data structures, and packet fields:

- Content popularity is tracked at the edge nodes in terms of locally perceived content request rate. Values are maintained in a *Popularity Table* and properly advertised in the new rate field of the Interest packet to account for the actual content request number over each edge link.
- Each node tracks the distance, in terms of hop count, from the on-path ingress nodes through a newly added field hopCount. This information, combined with the content request rate, is used to compute the PaC metric that is then advertised in the new pac field of the Interest packet by the forwarding nodes.
- The highest PaC metric, discovered during the Interest forwarding, is carried by the returning Data packet in a new pac field, and it is used to select the cacher.

**Figure 3:**
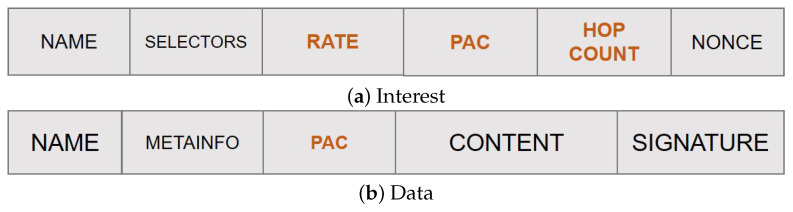
Overhauled NDN packets (fields with text in orange are those added by the PaC-based caching).

Figure 3 shows the new fields introduced in the Interest and Data packets, respectively, that enable the PaC-based caching thanks to an implicit signalling mechanism, which will be clarified subsequently.

### 3.2. Tracking Content Popularity

Similarly to other schemes [6,10,11,13], the edge nodes track in the Popularity Table the received requests in order to identify the most popular contents.

Basically, the ingress nodes (green devices in Figure 2) counts the Interests received from consumers they are connected to, in order to determine the average request rate of each content. At the ingress node $v_{j}\in I$, the average request rate for content $x_{n}$ is denoted as $R_{v_{j}}\left(x_{n}\right)$.

The node periodically updates the average request rate, with a time interval *T* set to one minute, similarly to [13], to properly infer potential changes in the request patterns. Therefore, we assume each entry in the Popularity Table includes three fields: content name, average request rate, and current counter of requests.

In our design, the ingress nodes are also in charge of identifying the most popular contents that should be cached along a delivery path. More specifically, a content is considered popular by the ingress node $v_{j}$ if it is requested a higher number of times than a popularity threshold $\Theta_{P}$. This latter is computed as the EWMA of the average request rate per each requested content:

(2) $$\Theta_{P}=\left(1-\alpha \right)\Theta_{P_{Old}}+\alpha \bar{R_{v_{j}}},$$

with

(3) $$\bar{R_{v_{j}}}=\frac{\sum_{n=1}^{M_{v_{j}}}R_{v_{j}}\left(x_{n}\right)}{M_{v_{j}}},$$

with $M_{v_{j}}$ being the number of distinct contents requested during the last time interval *T*, as perceived by node $v_{j}$, and α∈(0,1) set to 0.125 to avoid large fluctuations in the computation and give relevance to the historical values.

Because the Interest aggregation in the PIT hides the actual number of consumers requesting the same contents, a specific signalling mechanism is deployed to let intermediate nodes effectively track the request rate. More specifically, each time an ingress node $v_{j}$ forwards an Interest for content $x_{n}$ to the next on-path node $v_{i}$, it includes the average request rate information in the rate field. Of course, the same edge node $v_{i}$ can be in multiple shortest delivery paths towards the same content. For instance, node $v_{4}$ in Figure 2 is traversed by two shortest paths from the ingress nodes $v_{8}$ and $v_{9}$. Instead, node $v_{2}$ is traversed by three shortest delivery paths from the ingress nodes $v_{8}$, $v_{9}$, and $v_{10}$.

We denote as ${\hat{I}}_{i}\left(x_{n}\right)\subset I$ the set of ingress nodes forwarding Interests for content $x_{n}$ to $v_{i}$. In other words, $v_{i}$ belongs to the shortest paths connecting the ingress nodes belonging to ${\hat{I}}_{i}\left(x_{n}\right)$, which receive the requests from the consumers, and the egress node towards the origin source. Since NDN implements only on-path caching, $v_{i}$ is a candidate cacher for a content $x_{n}$ for which requests are received by ingress nodes in ${\hat{I}}_{i}\left(x_{n}\right)$. For instance, a content cached at $v_{2}$, in Figure 2, may serve the requests coming from the three ingress nodes, $v_{8}$, $v_{9}$, $v_{10}$.

The intermediate node $v_{i}$ (e.g., $v_{4}$ in Figure 2) collects the $R_{v_{j}}\left(x_{n}\right)$ values from the Interests received through the incoming interfaces and calculates the local average request rate as:

(4) $$R_{v_{i}}\left(x_{n}\right)=\sum_{j\in {\hat{I}}_{i}\left(x_{n}\right)}R_{v_{j}}\left(x_{n}\right).$$

When re-transmitting the Interest packet, $v_{i}$, in its turn, overwrites the rate field with the new cumulative value, thus the next-hop node will be aware of the average number of requests that can be satisfied with a Data packet over that incoming interface. The next-hop node (e.g., $v_{2}$ in Figure 2) also calculates the cumulative request rate, and so on.

### 3.3. Popularity-Aware Closeness Metric

The PaC metric of a potential cacher $v_{i}$ for content $x_{n}$, $PaC(v_{i},x_{n})$, considers the distance, in terms of hop count, between $v_{i}$ and the ingress nodes connected to the consumers. Since, in NDN, $v_{i}$ receives Interests for $x_{n}$ from an ingress node $v_{j}$ only if it is in the forwarding path between $v_{j}$ and the origin source, $PaC(v_{i},x_{n})$ takes into account only the set of ingress nodes ${\hat{I}}_{i}\left(x_{n}\right)$.

In parallel, the metric considers the number of consumers that could be satisfied by the potential cacher. The higher is the number of requests for $x_{n}$ that cross $v_{i}$, the higher should be the PaC metric.

In mathematical terms, the PaC metric for a node $v_{i}\in V$ and a content $x_{n}$ can be expressed as:

(5) $$PaC\left(v_{i},x_{n}\right)=\frac{R_{v_{i}}\left(x_{n}\right)\left|{\hat{I}}_{i}\left(x_{n}\right)\right|}{\sum_{j\in {\hat{I}}_{i}\left(x_{n}\right)}\left(h_{\left(v_{i},v_{j}\right)}+1\right)}$$

where $|{\hat{I}}_{i}\left(x_{n}\right)|$ is the cardinality of the set of ingress nodes in ${\hat{I}}_{i}\left(x_{n}\right)$, used to normalize the metric, and $h_{\left(v_{i},v_{j}\right)}$ is the hop distance between $v_{i}$ and the ingress node $v_{j}$.

It can be observed that $PaC(v_{i},x_{n})$ increases with the request rate of $x_{n}$ and it is equal to zero if $v_{i}$ does not receive any request for $x_{n}$. At the same time, the metric decreases if $v_{i}$ is far from the consumers.

### 3.4. Caching Algorithm

When an Interest for content $x_{n}$ arrives at an ingress node $v_{j}$, the latter updates the corresponding entry in the Popularity Table, checks if the content is popular, and then accesses the CS to find a matching Data packet to send back immediately. If the CS lookup fails, then $v_{j}$ checks the PIT. If this lookup also fails, then $v_{j}$ acts differently depending whether the content is popular, i.e., its average request rate is higher than the popularity threshold, or not. If it is not popular, then there is no need to cache it along the path. Therefore, $v_{j}$ increases by one the hopCount field of the Interest, fills the rate field, in order to allow the next hop updating the Popularity Table, but leaves the pac field to the default zero value, to indicate that the content is not popular. The request will be forwarded towards the origin server according to the standard NDN forwarding fabric. Of course, it may happen that an edge on-path node belonging to multiple delivery paths has cached the content (because it is considered popular by another ingress node) and, therefore, the request can be still satisfied at the edge.

The returning Data packet, being not popular, should not be cached. However, to make the best of the available storage space, unpopular Data packets can be cached in case the CS is not full, e.g., during the network bootstrap phases.

Vice versa, if $x_{n}$ is popular, then $v_{j}$ updates all the new fields of the Interests, i.e., hopCount, rate, and pac, and transmits the packet to the next-hop, according to the FIB entry.

The subsequent node $v_{i}$ receiving the Interest performs a slightly different processing. First, it accesses the information from the Interest header fields and checks if the Popularity Table needs to be updated. In NDN, Interests cannot loop, therefore a newly received Interest carries consistent information in the header fields. The latter can be equal to the previously recorded one, if the request pattern has not changed. The node then performs a lookup in the CS for a match. If the Data packet is found, then $v_{i}$ computes its PaC metric and compares it with the value in the pac field. If its value is greater, then it overwrites the field and sends the packet back. Vice versa, if its PaC value is smaller, it simply sends the Data packet without altering it. The receiving node with the highest PaC will also cache the Data, thus moving the copy closer to the consumers.

In case the CS and the consequent PIT matching fail, $v_{i}$ computes its PaC and, if its value is greater than the current one, it updates the pac, hopCount, and rate fields. Conversely, if the PaC is lower than the current one, it only updates the hopCount and rate fields. It then re-transmits the request according to the FIB and waits for the Data packet.

When finally receiving the Interest, the origin server, or an intermediate cacher, copies the PaC value from Interest to the corresponding field in the Data packet header and transmits the packet. The first node with the corresponding PaC will cache the Data. If the CS if full, an existing item is replaced according to the LRU policy.

**Figure 4:**
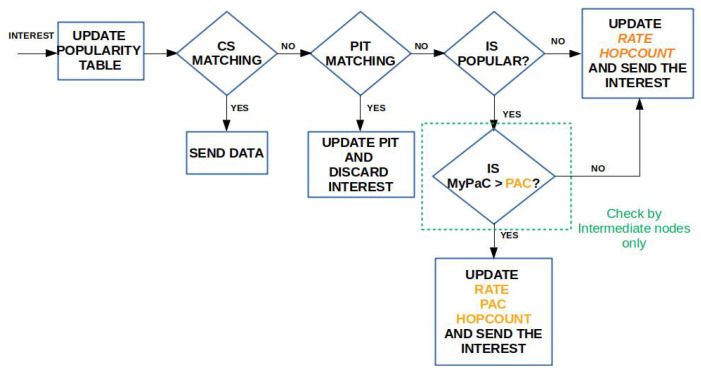
Interest processing in the presence of PaC-based caching.

**Figure 5:**
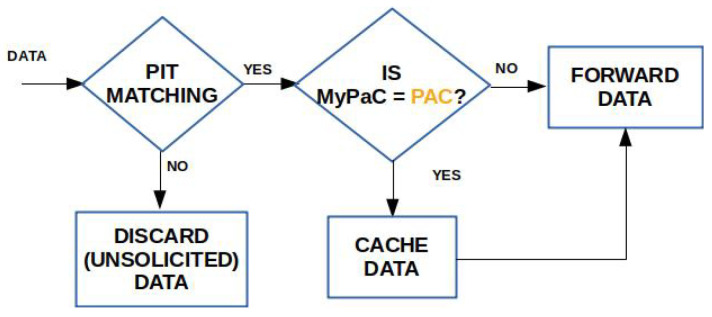
Data processing in the presence of PaC-based caching.

The flowcharts summarizing the Interest processing and Data processing for the PaC-based caching are depicted in Figure 4 and Figure 5, respectively.

### 3.5. A Toy Example

**Figure 6:**
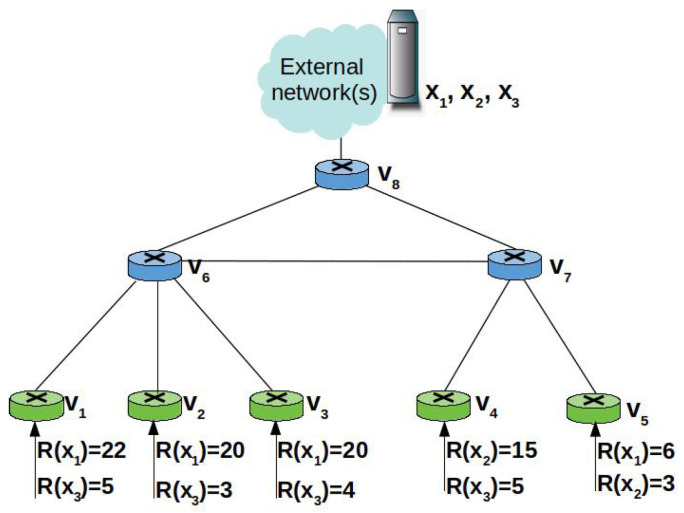
Toy example: an edge network with eight nodes.

To better understand the PaC metric, we consider the toy example in Figure 6 that includes eight edge nodes in a hierarchical topology. The nodes $v_{1}$, $v_{2}$, …,$v_{5}$ are ingress nodes tracking the average content request rate. For the sake of simplicity, we assume that three distinct popular contents, namely $x_{1},x_{2},x_{3}$, should be cached at the edge and their request rate is stable. The available storage space at each node allows for caching only one content.

It can be observed that the average request rate for $x_{1}$, the most requested content at the edge, is 22 at $v_{1}$ and 20 at $v_{2},v_{3}$, whereas it is 6 at $v_{5}$. When computing the PaC metric for the nodes traversed by the Interests for $x_{1}$, according to Equation (5), it results in $PaC(v_{1},x_{1})=22$, $PaC\left(v_{2},x_{1}\right)=PaC\left(v_{3},x_{1}\right)=20$, $PaC(v_{5},x_{1})=6$, $PaC\left(v_{6},x_{1}\right)=\frac{62\cdot 3}{6}=31$, $PaC\left(v_{7},x_{1}\right)=\frac{6\cdot 1}{2}=3$, and $PaC\left(v_{8},x_{1}\right)=\frac{68\cdot 4}{12}=22.66$. The node with the highest PaC metric for $x_{1}$ is $v_{6}$, which is indeed the node closer to the majority of consumers that are attached to the ingress nodes $v_{1}-v_{3}$. If instead we consider the path $\left\{v_{5}\to v_{7}\to v_{8}\right\}$, the node with the highest PaC is $v_{8}$. However, caching $x_{1}$ at $v_{6}$ would imply that the node at the upper layer, $v_{8}$, will not receive further Interests for $x_{1}$ and therefore, its local request rate will decrease. As a consequence, in a subsequent time window, $x_{1}$ will be cached at $v_{5}$.

Vice versa, an approach based on the betweenness centrality, like BEP, would be considered as best cacher for $x_{1}$ node $v_{8}$, which has the highest centrality, but it is also the farthest away from the consumers. Therefore, caching $x_{1}$ at $v_{8}$ would highly increase the retrieval delay and the intra-domain traffic.

Content $x_{2}$ is requested only at $v_{4}$ and $v_{5}$ and it results in $PaC(v_{4},x_{2})=15$, $PaC(v_{5},x_{2})=3$, $PaC\left(v_{7},x_{2}\right)=\frac{18\cdot 2}{4}=9$, $PaC\left(v_{8},x_{2}\right)=\frac{18\cdot 2}{6}=6$. The node with the highest PaC metric is $v_{4}$ which, again, is the one closer to the majority of consumers. Therefore, the majority of requests will be served with minimum delay and with minimum intra-domain traffic. To serve requests coming from path $\left\{v_{5}\to v_{7}\to v_{8}\right\}$, instead, $v_{7}$ would be selected as cacher.

By following the same procedure, it can be found that the higher PaC for content $x_{3}$ is obtained at $v_{8}$.

## 4. Performance Evaluation

### 4.1. Simulation Settings

The proposed caching strategy has been implemented in ndnSIM, the official simulator of the NDN research community [16]. As representative edge domain, we consider a tree topology randomly generated with the Georgia Tech Internetwork Topology Models (GT-ITM) [17]. The topology includes 20 intermediate nodes and 8 leaves acting as ingress nodes. The root node connects the edge domain with a remote server acting as content producer. Because the performance assessment is focused on the edge domain, the external network is simply simulated as a link, with latency of 30 ms [18], between the root node and the server. The latency of edge links is instead uniformly distributed in the range [2–5] ms.

We consider a catalog of 15,000 contents, each one consisting of 1000 Data packets 1 Kbyte-long. A variable number of consumers attached to the leaf nodes of the topology, request contents according to the Zipf’s law [3], with skewness parameter α set to 1. In these settings, we consider two distinct simulation scenarios.

- In the first scenario, we assume that the total caching capacity of the edge domain, uniformly distributed among the nodes, is varying from 0.25% to 2% of the overall catalog size, similarly to the values reported in [19]. The number of consumers is set to 60.
- In the second scenario, we assume that the cache capacity is fixed to 0.5% of the overall catalog size, whereas the number of consumers range from 20 to 70.

The main simulation settings are summarized in Table 3.

We compare the proposed model (labeled as *PaCC* in the plots) against the following benchmark solutions:

- Cache everything everywhere (labeled as CEE in the plots). It is the vanilla NDN caching strategy where all the incoming Data packets are cached.
- Fixed probability-based caching (labeled as *Prob* in the plots). It caches incoming Data according to a fixed probability set to 0.5 [9].
- Betweenness and edge popularity caching (labeled as BEP in the plots). It implements a caching scheme based on the popularity-aware betweenness centrality metric, as in [6].

For all the noted schemes, the replacement policy is LRU.

The following performance metrics are considered:

- Retrieval delay: it is computed as the average time taken by a consumer to retrieve a content.
- Number of hops: it is computed as the average number of hops traveled by the Interest packets for retrieving the corresponding Data packets.
- Exchanged NDN packets: it is the total number of Interest and Data packets transmitted by all the nodes, i.e., consumers, providers, and edge nodes, during the simulation, to retrieve the contents.

The first two metrics capture the effectiveness of the compared schemes in caching content copies in proximity to the consumers. The last metric provides insights about the efficiency of the schemes, in terms of traffic exchanged in the network. Results are averaged over 10 runs and reported with 95% confidence intervals.

### 4.2. Results

#### 4.2.1. Impact of the Cache Size

**Figure 7:**
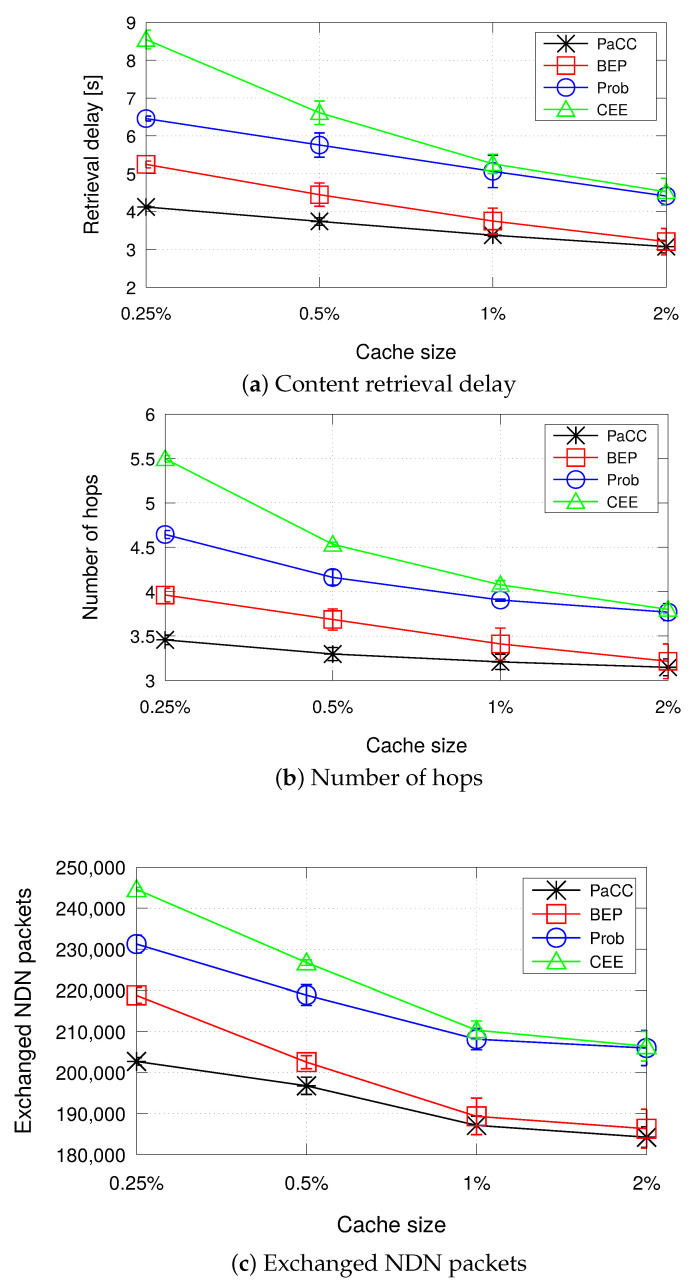
Metrics when varying the cache size of the edge domain from 0.25% to 2% of the content catalogue size (number of consumers equal to 60).

Figure 7 shows the performance metrics when varying the cache size of the edge domain from 0.25% to 2% of the content catalogue size. As expected, it can be observed that, as the cache size increases, performance improves for all the considered schemes. Indeed, the higher the storage space at the edge, the higher is the number of contents that can be stored, with consequent advantages in terms of reduced retrieval delay, number of hops, and exchanged traffic.

Being oblivious of content popularity and topology information, the simplest CEE solution shows the worst performance in terms of content retrieval delay, due to higher number of hops traversed to reach the requested content. It generates the highest load of exchanged NDN packets. The Prob scheme slightly outperforms CEE, because it does not cache indiscriminately contents but it tries to better distribute them in the CS of edge nodes.

As the cache size of the edge domain increases, differences among the two decrease, because there is higher chance to find storage space for caching contents within nodes.

PaCC outperforms all the other schemes in terms of all the considered metrics. However, the gap with BEP reduces as the edge domain is more capable to cache contents.

**Figure 8:**
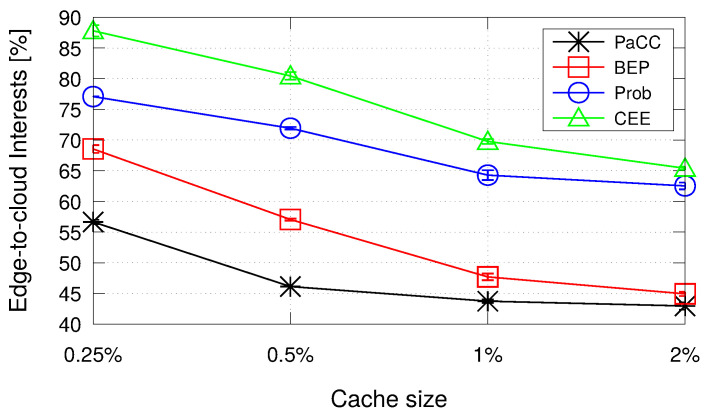
Percentage of Interest packets that are forwarded to the origin server.

To conclude the analysis, Figure 8 shows the percentage of Interest packets that reach the origin server because it has not been found a CS matching at the edge. Reasonably, the traffic to the cloud reduces when the storage space at the edge increases, with PaCC outperforming all the benchmark schemes.

#### 4.2.2. Impact of the Number of Consumers

**Figure 9:**
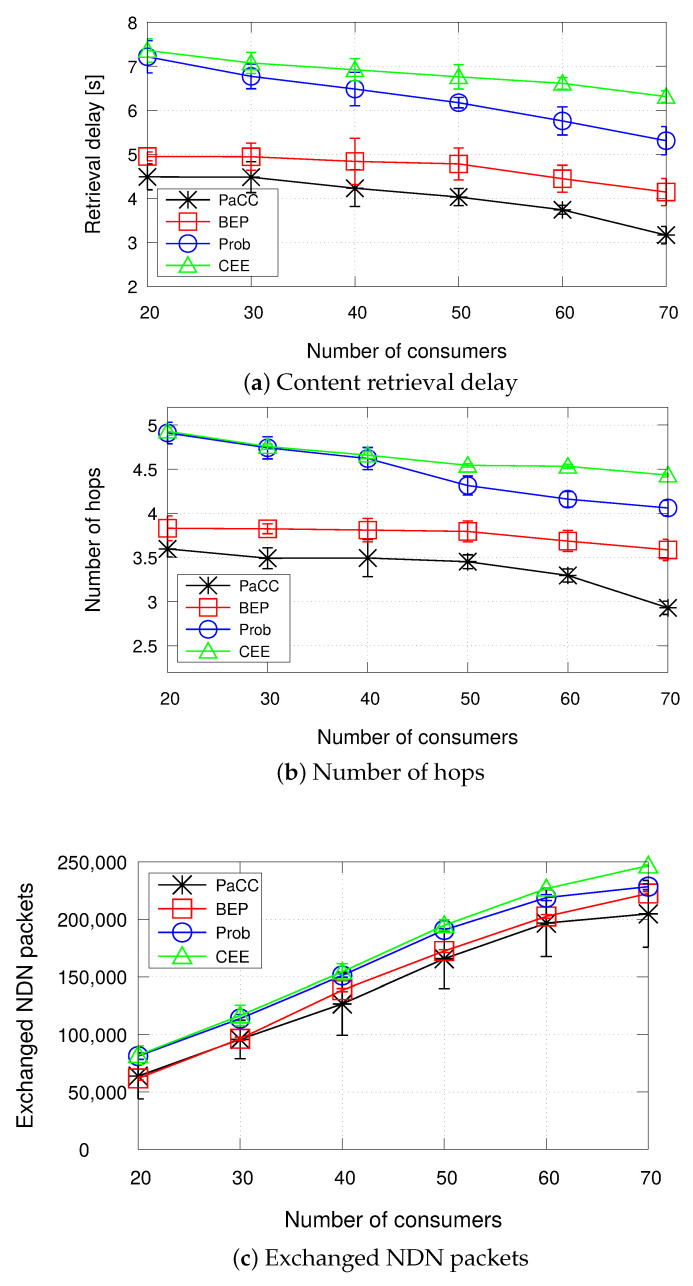
Metrics when varying the number of consumers (cache size equal to 0.5% of the catalogue size).

The second set of results, reported in Figure 9, measures the metrics of interest when varying the number of consumers.

Under such settings, the proposed PaCC solution achieves the best performance in terms of content retrieval delay, number of hops, and exchanged NDN packets.

It can be observed that, as the number of consumers increases, all schemes experience a shorter delay and a lower number of hops. Such a behaviour has to be ascribed to the fact that under the simulated settings, due to the Zipf distribution, a higher number of requests, from different consumers, concentrate on the same few contents, the most popular ones. Hence, such contents are more likely to be cached in the edge domain, instead of being retrieved from the original producer. The number of exchanged packets, instead, reasonably increases with the number of consumers, to account for the increasing number of Interests issued by more consumers and number of Data packets forwarded to each single consumer.

#### 4.2.3. Overhead Analysis

To better show the pros and cons of the conceived solution, we summarize in Table 4 the main differences between the compared schemes in terms of incurred overhead, i.e., additional signalling bytes piggybacked in the exchanged NDN packets. A high signalling overhead introduced by a caching strategy could deteriorate the content retrieval performance, e.g., by increasing the content retrieval delay due to the transmission of larger packets that occupy the channel for a longer time and may generate a higher channel load and resulting congestion. It is worth observing that the blind CEE and Prob schemes incur no additional overhead. Similarly to PaCC, BEP foresees one additional field in the Data packet to convey the maximum betweenness to be compared with that of the nodes along the backforwarding path. It is the PaC metric in our proposal. Three additional fields per Interest are foreseen by PaCC, for a total of 10 bytes (less than 1% overhead per packet). For BEP, the overhead per Interest is a function of the number of nodes traversed along the path (|Π|) by the packet carrying the BetweennessArray field. For a path made of 5 nodes the overhead gets equal to 24 bytes. Under the majority of settings (|Π|>1), PaCC is also more efficient than BEP in terms of exchanged bytes in the network per packet. Therefore, the signalling overhead introduced by PaCC is extremely low, which contributes to limit the retrieval delay metric, as showed in Figure 7 and Figure 9.

## 5. Conclusions

In this work we have proposed a novel caching strategy for edge domains, called PaCC, which accounts for content popularity and proximity to the consumers. Achieved results, collected under a variety of settings, confirm that the devised solution allows more judicious content caching decisions that are particularly crucial when the storage capabilities of the edge domain are small. As a consequence, the content retrieval delay experienced by PaCC is reduced compared to the considered benchmark schemes.

As a further benefit, the proximity of contents to consumers achieved by PaCC allows for reduction in the overall amount of exchanged data traffic at the expense of a negligible additional overhead per NDN packet. Such a finding is relevant because future networks will be overwhelmed by a myriad of (huge) contents exchanged by massively deployed devices and requested by increasingly demanding users.

The performance of the conceived solution can be further improved by addressing the following aspects: (i) optimizing the method for tracking the content popularity, (ii) optimizing the computation of the popularity-aware closeness metric. In PaCC, a threshold-based mechanism is implemented to track the popularity of contents based on the number of received content requests. However, more accurate mechanisms could be introduced in our design, for instance based on artificial intelligence (AI) content popularity prediction algorithms [20]. In parallel, more accurate metrics could be considered to estimate the proximity of the cachers to the consumers. In our design, we leverage the hop count metric, which, however, cannot reflect the presence of congested links or congested nodes. The proximity information could be improved by taking into account other additional metrics like the round-trip-time over the network links and/or the load on the nodes.

## Figures and Tables

**Table 1:** Comparison of caching strategies based on popularity and/or topology metrics.

Work	Popularity	Topology	Domain	Decision Strategy
[10]	✓	-	Edge/Core	Caching popular contents based on a fixed popularity threshold
[11]	✓	-	Edge	Caching popular and fresh contents based on a flexible popularity threshold
[12]	✓	-	Edge/Core	Caching only popular long-lasting contents (in the core network); caching popular short-lasting contents only once per each delivery path (in the edge network)
[13]	✓	-	Edge/Core	Caching popular contents based on a flexible popularity threshold
[14]	✓	-	Edge	Caching popular contents based on a strict hierarchical coordination between the nodes
[4]	-	✓	Edge/Core	Caching contents in the most central nodes based on the betweenness centrality metric
[5]	✓	✓	Edge	Caching based on a popularity-weighted content-based centrality
[6]	✓	✓	Edge/Core	Caching based on popularity and betweenness centrality metric
Our work	✓	✓	Edge	Caching based on a popularity aware consumer proximity metric

**Table 2:** Summary of the main notations.

Symbol	Description
*V*	set of NDN edge nodes
*I*	set of NDN ingress nodes, with I⊂V
*X*	catalogue of contents
$v_{j}$	generic ingress node
$v_{i}$	generic edge node
$x_{n}$	generic content
$R_{v_{i}}\left(x_{n}\right)$	average request rate for content $x_{n}$ at node $v_{i}$
$\Theta_{P}$	popularity threshold
$\bar{R_{v_{j}}}$	average content request rate at ingress node $v_{j}$
*T*	time interval for updating caching decision parameters
$M_{v_{j}}$	number of distinct contents received at the ingress node $v_{j}$
${\hat{I}}_{i}\left(x_{n}\right)$	set of ingress nodes forwarding Interests for content $x_{n}$ to node $v_{i}$
$PaC(v_{i},x_{n})$	popularity-aware closeness metric for content $x_{n}$ at node $v_{i}$
$h_{\left(v_{i},v_{j}\right)}$	hop distance between nodes $v_{i}$ and $v_{j}$

**Table 3:** Main simulation settings.

Parameter	Value
Content catalog size	15,000 contents
Content size	1000 Data packets
Data packet size	1000 bytes
Content Popularity	Zipf-distributed with α = 1
Scenario	GT-ITM [17]
Edge link latency	Uniformly distributed in [2, 5] ms
Number of consumers	20–70
Number of edge nodes	28
Caching capacity	From 0.25% to 2% of the catalogue size

**Table 4:** Additional fields per packet: PaCC vs. benchmark schemes.

Strategy	Interest (Size in Bytes)	Data (Size in Bytes)
CEE	-	-
Prob	-	-
BEP	PopularityRanking (4)	Betweenness (4)
	BetweennessArray (4×|Π|)	
PaCC	Rate (4)	Pac (4)
	Pac (4)	
	HopCount (2)	

## Data Availability

Not applicable.
